# Who receives an early or timely diagnosis? Data from DETERMIND baseline

**DOI:** 10.1002/alz70860_106345

**Published:** 2025-12-23

**Authors:** Ben Hicks

**Affiliations:** ^1^ Brighton and Sussex Medical School, Brighton, United Kingdom

## Abstract

**Background:**

The evidence base concerning the socio‐demographic factors associated with diagnostic timing is limited and studies often use traditional regression methods, focussing solely on direct associations and ignoring those indirect pathways; thereby simplifying the links and missing potentially informative associations. No studies to date have sought to map out the direct and indirect factors associated with an early and timely diagnosis of dementia.

**Methods:**

DETERMIND consists of 940 newly diagnosed people with dementia, and their 698 carers, across three diverse areas of England. Drawing on baseline data we created multiple study‐designed indices of early diagnosis using severity of dementia at diagnosis (cognitive) or time between self‐reported first signs of symptoms and clinically reported diagnosis date (temporal). ‘Timely’ diagnosis indices used self‐report items from people with dementia and their carers comparing those who reported the diagnosis was ‘made at the right time’ to those who believed it was ‘too early’ or ‘too late.’ Complex pathway analysis enabled us to investigate the direct and indirect associations between background variables and our indices of early and timely diagnosis.

**Result:**

Our findings suggested numerous and sometimes contradictory effects of socio‐demographic determinants on the direct and indirect pathways to diagnosis and these varied depending on the definition of diagnostic timing being examined. Cognitively early diagnosis was more frequent in care dyads who were white, whereas temporally early diagnosis was more frequent in people with dementia who were not white. Our findings also highlighted different determinants associated with an early vs *timely* diagnosis of dementia.

**Conclusion:**

Using complex pathway analysis we have generated unique insights into the socio‐demographic determinants associated with an early vs late and timely vs untimely diagnosis. Our findings highlight the importance for clearly operationalising ‘early’ diagnosis to compare findings across studies and generate a comprehensive understanding of factors associated with diagnostic timing. Furthermore, we suggest early diagnosis does not necessarily equate to ‘timely’ diagnosis and so these concepts cannot be used interchangeably in research or in policy.

